# Secure biosystems design in *Saccharomyces cerevisiae* establishes effective biocontainment strategies and mechanisms of escape

**DOI:** 10.1128/aem.00741-25

**Published:** 2025-08-13

**Authors:** Natalie A. Lamb, Kimberly A. Rosenbach, Thomas J. Musselwhite, Kathleen L. Arnolds, Riley C. Higgins, Gabriella A. Li, Jeffrey G. Linger, Michael T. Guarnieri

**Affiliations:** 1 National Renewable Energy Laboratory, Renewable Resources and Enabling Sciences Center, Golden, Colorado, USA; 2 National Renewable Energy Laboratory, Biosciences Center, Golden, Colorado, USA; 3 Department of Chemical and Biological Engineering, Colorado School of Mines 3557, Golden, Colorado, USA; 4 Renewable & Sustainable Energy Institute, University of Colorado, Boulder, Colorado, USA; University of Illinois Urbana-Champaign, Urbana, Illinois, USA

**Keywords:** escape mechanisms, genetically modified microorganisms, bioproduction, whole genome sequencing, yeast genetics, secure biosystems, biotechnology, biocontainment

## Abstract

The widespread application of recombinant DNA and synthetic biology approaches for microbial metabolic engineering pursuits has motivated the development of biocontainment strategies, targeting safe and secure deployment of genetically modified microorganisms (GMMs). However, the design rules and mechanistic drivers governing biocontainment efficacy, as well as impacts of biocontainment upon microbial fitness, remain to be comprehensively evaluated, hindering predictive design and application of these strategies. We have developed a platform for high-resolution analysis of a transactivated kill switch in laboratory and industrial strains of *Saccharomyces cerevisiae* to assess modes of biocontainment escape and establish design rules for development of kill switch systems in diverse microbes. A camphor-regulated, RelE toxin system was systematically deployed to assess the impacts of differential kill switch copy number and ploidy in laboratory vs industrial strains. CRISPR-mediated integration of the biocontainment system at various loci revealed rapid escape events driven, in part, by mutations to both the Cam-transactivator (cam-TA) and RelE toxin. Genetic engineering enabled recapitulation of escape phenotypes, confirming mechanisms of escape and establishing structure-function relationships in the cam-TA system. Interestingly, genomic resequencing of escape mutants also revealed a series of off-target mutations, implicating additional modes of kill switch escape. Multi-copy integration of the kill switch system mitigated these effects by orders of magnitude, without compromising the biosynthetic capacity of the microbes, but proved insufficient to establish sustained biocontainment. The resultant data define a series of key design rules for next-generation biocontainment strategies and add to a growing foundational knowledge base targeting establishment of secure biosystems designs.

**IMPORTANCE:**

The development of biocontainment mechanisms is essential for safe deployment of microbes in industrial processes and to minimize escape into the natural environment. To achieve secure biosystems designs, a deeper understanding of the mechanistic drivers governing biocontainment efficacy and associated impacts of biocontainment upon microbial fitness are needed. This study uncovers successful biocontainment strategies for kill switch deployment in addition to mechanistic information conferring kill switch escape. Additionally, differential effects were observed in laboratory vs industrial yeasts, implicating auxotrophies and heterothallism as additional drivers of biocontainment. These learnings can inform iterative designs, with the goal of improving the efficacy of biocontainment while maintaining fitness in engineered microbes.

## INTRODUCTION

The rapidly expanding bioeconomy is dependent on the development of genetically modified microorganisms (GMMs) for the production of bioproducts and biochemicals (1–4). Microorganisms are also engineered for deployment in bioremediation, biomining, and other environmental release contexts (5–8). Incorporating secure biosystems design into GMMs is essential to maximize biocontainment and minimize impacts on surrounding environments in either intentional or unintentional release scenarios, through the release of a GMM or its genetic material into the environment (9–12). Underscoring the importance of biocontainment, the NIH has released standards establishing a target escape frequency of 10^−8^ for GMMs (13). To meet NIH biocontainment standards, it is essential to establish robust biocontainment safeguards and to understand mechanisms by which microorganisms circumvent these strategies. A loss of regulation from biocontainment and growth in non-permissive conditions is herein defined as “escape.” Uncovering mechanisms that contribute to escape provide critical knowledge that can inform iterative biocontainment designs. In addition, it is critical to ensure that biocontainment does not impact biocatalytic productivity, especially in the context of GMMs engineered to produce biomolecules at scale.

In response to the need for secure biosystems designs, efforts to develop new biocontainment strategies have greatly expanded over the last 10 years (14). One of the most well-established forms of biocontainment is genetic auxotrophies (15, 16), whereby the organism is dependent on the supplementation of an exogenous compound in order to survive. Auxotrophy as a biocontainment strategy has been further expanded to rely on xeno molecules, such as nonstandard amino acid (nsAA) supplementation for survival (17, 18). Kill switches are another foundational biocontainment method, which have been successfully deployed across a number of microbial hosts (19–21). Kill switches rely on a mode of lethality artificially encoded into the host organism, and death is triggered by the presence or absence of an external molecule or stimulus. Kill switches have been widely deployed across diverse microbes and are highly efficacious for containing GMMs, yet the principles governing kill switch efficacy have yet to be fully determined.

The budding yeast, *Saccharomyces cerevisiae,* is a powerful system for understanding the intricacies of both biocontainment and bypass thereof; escape. *S. cerevisiae* has an expansive genetic toolkit and is used in industry as a microbial chassis for the production of a myriad of food, beverages, biopharmaceutical products, and chemicals including ethanol and other biofuel precursors (22–25). Many novel containment strategies have been developed in *S. cerevisiae*, including those that exploit synthetic lethality, protein degradation, and non-standard amino acids (17, 18, 26, 27). Furthermore, auxotrophies are present in most laboratory strains of yeast, while industrial strains grown at scale often lack genetic auxotrophies (28, 29). This discrepancy is primarily attributed to the cost of adding media components to compensate for auxotrophies, along with evidence that auxotrophic deficiencies can impair productivity and reduce resilience to stressors, even when strains are externally supplemented with nutrients to complement auxotrophies. Kill switches have also been utilized as a means of biocontainment in *S. cerevisiae* (30), but have yet to be reported in industrial strains of yeast, and limited research has described the effects of kill switches on biocatalytic productivity (9, 14, 29). Accordingly, the development of efficacious biocontainment in industrially relevant strains is critical for developing secure biosystems design strategies that are translatable.

**Fig 1:**
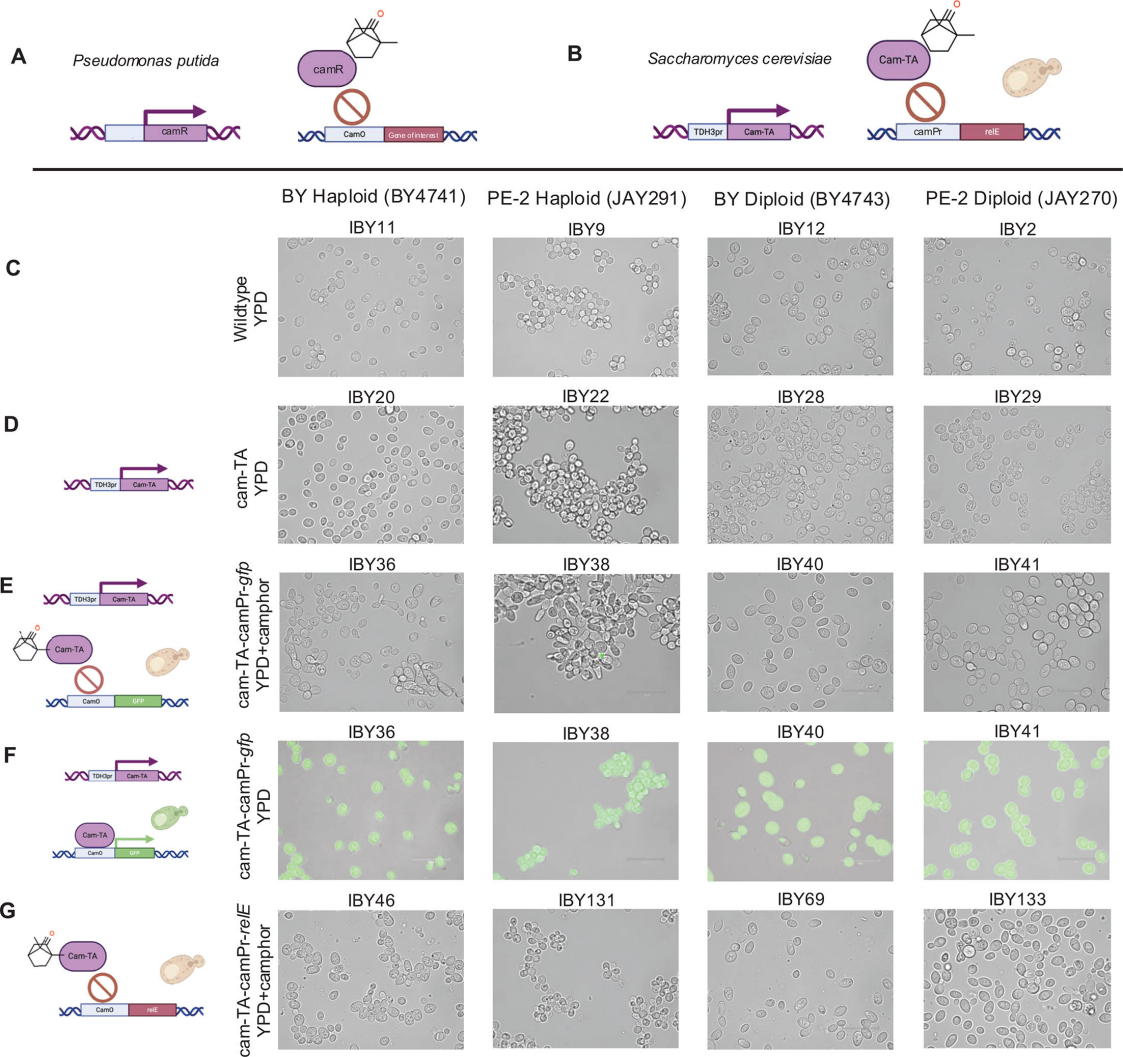
The CamOff-RelE system effectively regulates GFP expression. (**A**) Representative image of the camphor oxidation operon from *Pseudomonas putida*. (**B**) A visual of the CamOff-RelE system developed in *S. cerevisiae*. *TDH3*pr-cam-TA is integrated on chromosome XII and camPr-*relE* is integrated on either Chr. X or XI. (**C**) Wildtype cells grown on YPD from the four different parent strain backgrounds including both BY and PE-2 haploids and diploids. (**D**) Cell morphologies with the addition of *TDH3*pr-cam-TA. (**E**) *TDH3*pr-cam-TA and camPr-*gfp* strains grown on camphor (**F**) *TDH3*pr-cam-TA and camPr-*gfp* strains fluoresce in the absence of camphor (**G**) CamOff-RelE strain morphology in the presence of camphor. Created in BioRender. Lamb, N. (2025) https://BioRender.com/n07x052

In this study, we paired the Camphor-Off (CamOff) system (31) with the endoribonuclease toxin RelE as a novel kill switch to elucidate underlying genetic factors that contribute to kill switch efficacy in both lab and industry strains of yeast. The CamOff inducible system is based on natural transcriptional regulation of the Cam Repressor in *Pseudomonas putida* by the small molecule camphor and was previously adapted in yeast (31) (Fig. 1A). Camphor is an aromatic, terpene ketone naturally found in the bark of the camphor tree but is now synthesized abundantly and inexpensively (31–34). RelE is a type II endoribonuclease toxin that acts by cleaving actively translating mRNA (35, 36) and has been shown to be lethal when expressed in *S. cerevisiae* (30). In laboratory conditions, CamOff-RelE yeast were grown in the presence of micromolar concentrations of camphor, maintaining the kill switch in an “off” state and preventing expression of the RelE toxin through inhibitory binding of the Cam-transactivator (cam-TA) (Fig. 1B). In the absence of camphor, the CamOff switch turns “on”; cam-TA binds to the Cam Operator sequence upstream of RelE, activating toxin expression and resulting in cellular lethality.

The CamOff-RelE system was integrated into both S288c lab strains of *S. cerevisiae* (BY4741, BY4743) as well as strains utilized in the Brazilian ethanol industry (PE-2 derivatives: JAY291, JAY270) (37, 38)*,* with the goal of creating a broadly efficacious biocontainment system and understanding underlying genetic factors, such as ploidy, auxotrophy, and genetic variability that impact patterns of biocontainment and escape. We hypothesized that increasing *relE* copy number under control of the CamOff system would increase biocontainment efficacy. Indeed, diploid strains containing two homozygous copies of CamOff-RelE were effectively contained with minimal cost to fitness and biocatalytic productivity. However, the industrial PE-2 strain had a higher escape rate than the lab strain BY, indicating underlying strain differences including the auxotrophies present in BY and heterothallism in PE-2 may play a role in biocontainment escape (37–40). Rare escape events in diploid organisms were observed via high-throughput screening and escape assays. Whole-genome sequencing of escapees revealed multiple genotypically distinct mechanisms of escape. Furthermore, mutation recapitulation showed that a single point mutation in cam-TA is causative in escape from the CamOff-RelE system. These findings elucidate mechanisms that lead to escape from the CamOff-RelE kill switch in *S. cerevisiae* and highlight fundamental considerations in biocontainment module design that can be applied to other organisms and biocontainment strategies.

## RESULTS

### The CamOff switch is functional in both lab and industrial strains of *S. cerevisiae*

The CamOff switch is modeled after the camphor oxidation operon from *Pseudomonas putida,* where a Cam Repressor (CamR) binds to the Cam Operon (CamO) sequence, activating downstream gene expression (31, 41, 42). In the presence of the small molecule camphor, CamR dissociates from, or is unable to bind to the CamO sequence (Fig. 1A). Previous work in *S. cerevisiae* designed the CamR-based transcriptional regulator (cam-TA), which is integrated under a constitutive yeast promoter (31). The Cam promoter (camPr) containing CamO sequence is integrated upstream of a gene of interest. Much like in *P. putida*, camphor supplementation prevents the binding of cam-TA to the camPr sequence in yeast, resulting in a loss of downstream gene expression (Fig. 1B). In this study, cam-TA was cloned under the constitutive yeast promoter, *TDH3*pr*,* in a Marker-Free CRISPR integration vector that targets Chromosome XII (Tables S1 to S3) (43). Either green fluorescent protein (*gfp*) or the toxin *relE* was added downstream of the camPr sequence in CRISPR integration vectors with homology for either Chromosome X or XI (Fig. 1;Tables S1 and S2). The CamOff-RelE containment system was integrated into the S288c derived BY series of yeast and industrially relevant PE-2 strains (Table S1). The BY series of *S. cerevisiae* are S288c derived strains that are well characterized and have known auxotrophies (39). BY4741 is a MATa haploid strain, with methionine, histidine, leucine, and uracil auxotrophies, and BY4743 is a diploid strain, heterozygous for the methionine auxotrophy and an additional lysine auxotrophy (39). PE-2 is a highly utilized prototrophic industry strain that is heterothallic and produces ethanol at high titers, rates, and yields (37, 38, 40). The addition of cam-TA to chromosome XII did not impact cell morphology in BY or PE-2 backgrounds (Fig. 1C and D) and strains grew at a rate commensurate with wildtype strains in liquid culture (Fig. S1). However, light microscopy revealed that cam-TA strains grown in the presence of camphor showed elongated morphologies (Fig. 1E). This is a well-documented growth effect, as camphor has been shown to affect chromosome condensation in *E. coli* (44) and also cause lengthened cell morphologies in *S. cerevisiae* (45). Despite altered cell morphology, minimal costs to growth rate were observed when wildtype strains were grown in the presence of camphor (Fig. S1).

To evaluate if the CamOff regulatory system effectively controlled gene expression in the presence and absence of camphor, *gfp* was integrated under control of camPr at Chromosome XI (Table S1). In the presence of camphor, cam-TA-camPr-*gfp* strains showed minimal fluorescence, indicating effective regulation of target gene expression (Fig. 1E). However, rare individual cells were seen that expressed GFP (Fig. 1E, PE-2 Haploid). Growth was evaluated in liquid culture to determine if there was any decrease in fitness upon addition of *gfp* under control of camPr. In strains with camPr, cam-TA is expected to bind to the camPr in the absence of camphor. Minimal costs to fitness were also observed in strains that had both cam-TA and camPr-*gfp* (Fig. S1). Although wildtype strains had little to no change in growth rate when grown with camphor, there was an impact on max OD rate and lagtime in cam-TA-camPr-*gfp* strains despite only modest changes in endpoint OD600 over the course of 72 hours (Fig. S1). In the absence of camphor, cells effectively expressed GFP, demonstrating the on/off switch effectively regulated gene expression (Fig. 1F). While previous work evaluated the system in the BY haploid, BY4741 (31), all cam-TA-camPr-*gfp* strains successfully turned on and off GFP expression in haploids and diploids, in both BY and PE-2 strain backgrounds (Fig. 1E and F). In liquid culture, GFP expression was stable over the course of seven days (data not shown), indicating effective long-term function of the cassette.

In addition to quantifying fitness, mutation rates in CamOff strains were determined. The impact of biocontainment construct integration on mutation rate is a crucial consideration for biocontainment design, as even a marginal increase in mutation rate could be a driver of escape through mutation accumulation, or associated genome instability could be detrimental for bioproduction process stability. A canavanine resistance assay was utilized to determine mutation rates at the *CAN1* gene and assess if camphor or the CamOff system had any impact on mutation rate in yeast (46–48). Assessing mutation rates at the *CAN1* is a widely used assay in both *S. cerevisiae* and *Schizosaccharomyces pombe* as an estimate for mutation rates genome wide (49–53). Mutation rates were determined in our suite of BY haploid strains, with and without biocontainment, grown in the presence and absence of camphor (see Materials and Methods). Notably, we did not observe a significant fold-change in wildtype BY strains grown with or without camphor (Table S4). This is consistent with previous work that found camphor does not increase mutagenesis in a microsome assay in *Salmonella typhimurium* (54). BY strains with cam-TA and cam-TA-camPr-*gfp* did not increase mutation rates above the wildtype BY4741 mutation rate. The fold change from the BY4741 wildtype ranged from 1.06 to 0.751, with overlapping confidence intervals, which suggests there was little to no impact on mutation rate from the addition of the CamOff system or growth in the presence of camphor (Table S4).

### Diploid CamOff-RelE strains are contained with minimal cost to growth and productivity

**Fig 2:**
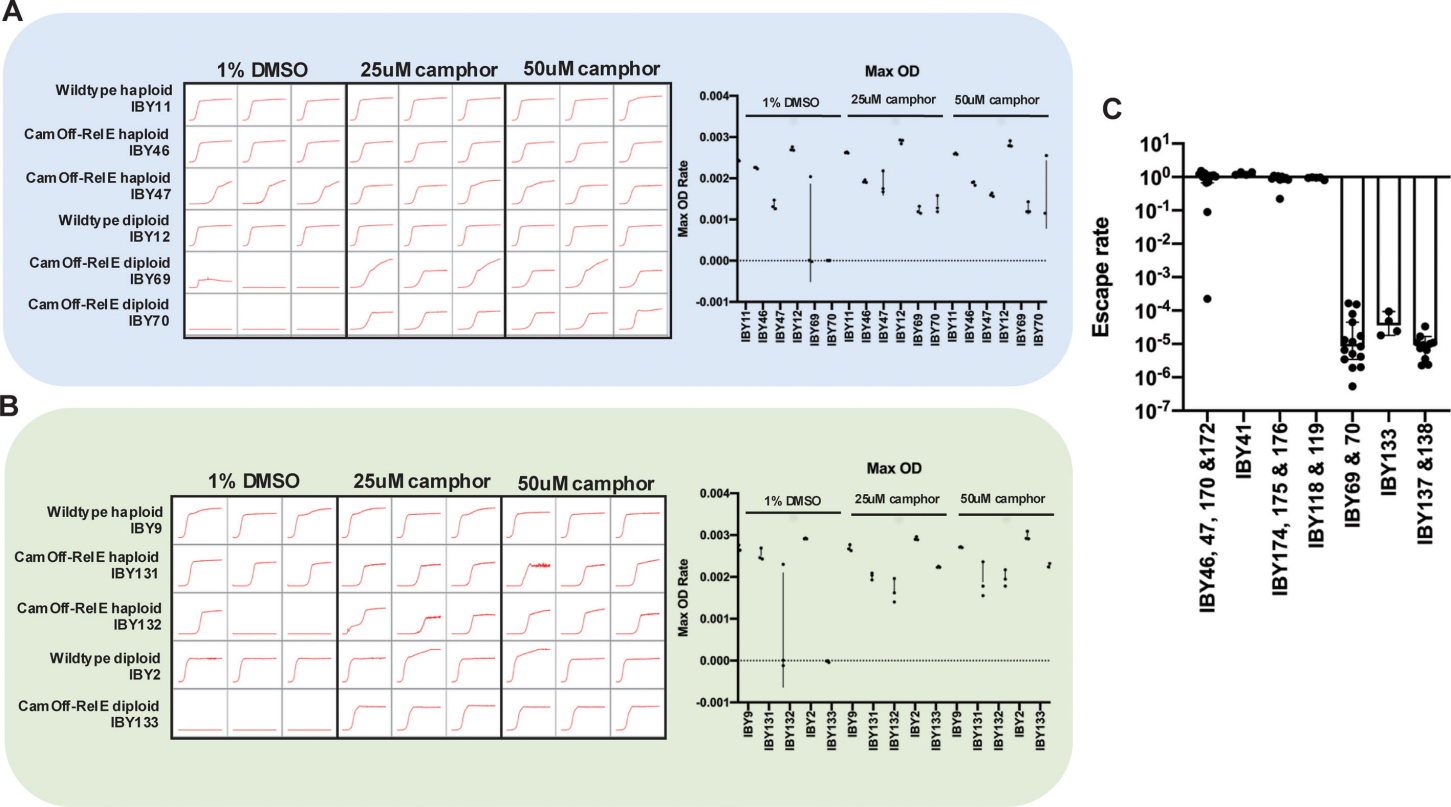
Escape screening shows containment in CamOff-RelE diploids. (**A**) Growth screening of BY-series of strains in liquid culture over 72 h. (**B**) Growth screening of PE-2 series of strains with CamOff-RelE. (**C**) Escape assays to quantify growth via plating assay for CamOff-RelE in both haploid and diploid strain backgrounds (see Table S1 for strain genotypes).

Biocontainment strains were constructed by adding the bacterial toxin gene *relE* behind camPr in strains that contained cam-TA (Fig. 1B; Tables S1 to S3). The cam-TA-camPr-*relE* kill switch was integrated in both haploid strains BY4741 and JAY291, as well as in diploid strains BY4743 and JAY270. CRISPR transformation led to homozygous integration of two copies of both cam-TA or camPr-*relE* in diploid strains, as previously described (43). Successful transformants were initially screened by a lack of growth in the absence of camphor, indicating effective containment. CamOff-RelE cells showed normal morphology when grown in the presence of camphor (Fig. 1G), with modest elongated morphologies as seen in Fig. 1E. We hypothesized that increasing the copy number of both cam-TA and camPr-*relE* would increase biocontainment efficacy, as prior reports showed a decrease in escape rate in diploid strains of yeast with the addition of transcriptional safeguards (26). Biocontainment efficacy was first assessed in cam-TA-camPr-*relE* strains with one copy (haploids) and two homozygous copies (diploids) of the containment system by monitoring growth in liquid YPD, with and without camphor (Fig. 2). In haploid CamOff-RelE strains, growth was observed in YPD both with and without the addition of camphor, indicating escape from the containment system. However, minimal growth was seen in the control condition (YPD + DMSO) over the course of 72 h in both BY (Fig. 2A) and PE-2 (Fig. 2B) diploid cam-TA-camPr-*relE* strains, indicating effective biocontainment.

**Fig 3:**
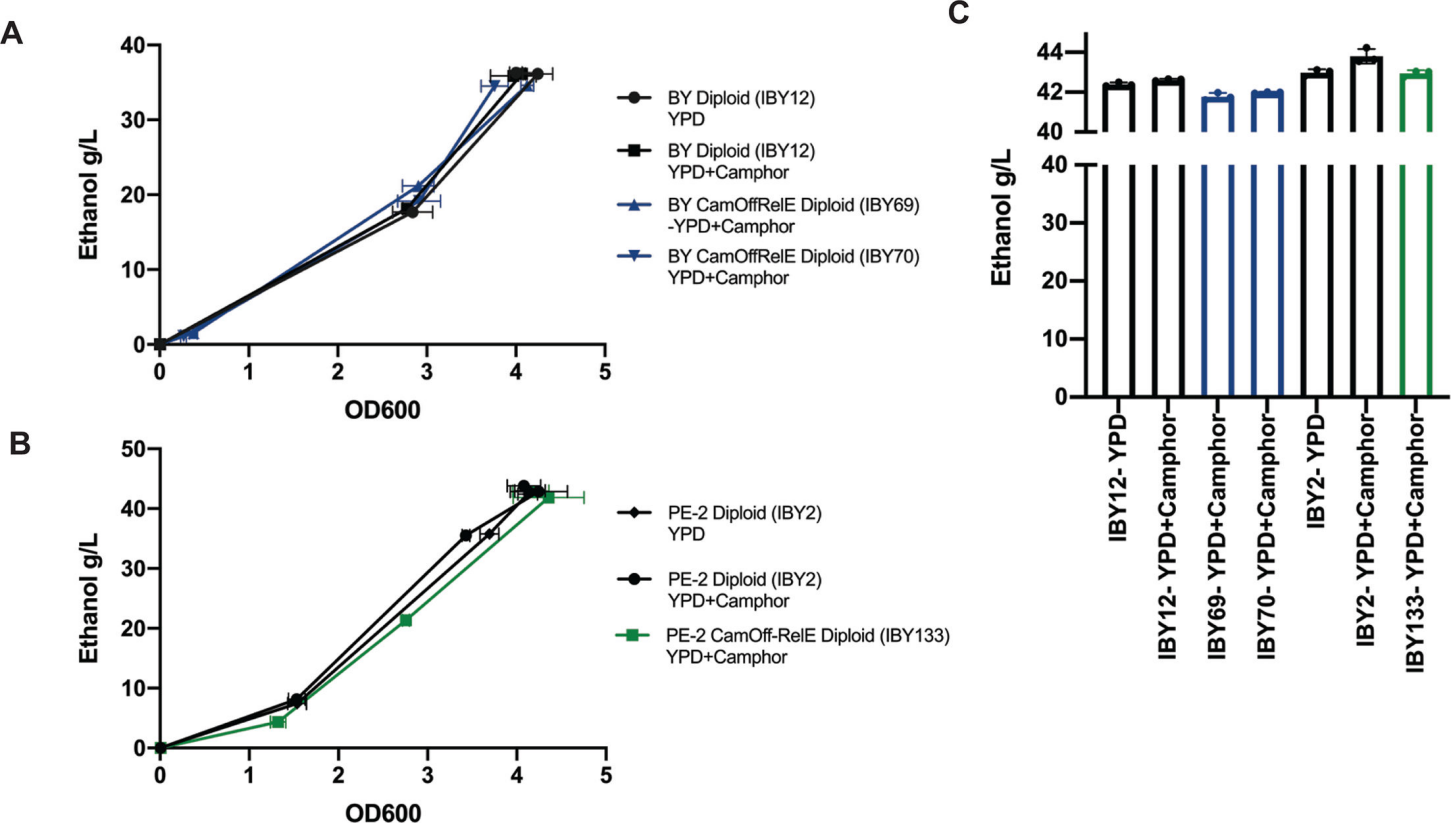
Ethanol production is maintained in biocontainment strains. (**A**) Ethanol quantification in diploid BY wildtype with and without Camphor and CamOff-RelE strains grown in the presence of camphor. (**B**) Ethanol quantification in the diploid PE-2 wildtype and biocontained strains. (**C**) After 5 days growth in anaerobic bottles, ethanol was quantified and reported in g/L.

To quantify the containment observed in diploid cam-TA-camPr-*relE* strains in liquid culture, escape rates were measured through a reversion assay, similar to previous work, by quantifying cells able to grow in the absence of Camphor (26). The escape assay allowed for long-term monitoring with precise quantification of escape frequency. In this assay, CamOff-RelE containment strains were grown in YPD with camphor and then plated under selective (YPD alone) and permissive conditions (YPD with 50 µM camphor). Colonies were counted under both selective and permissive conditions, and the method of the median was used to determine escape frequency (26, 55) (see Materials and Methods). The BY diploid CamOff-RelE strain with two copies of the containment system had a median escape rate of 8.33 × 10^−6^, and the PE-2 diploid had a median escape rate of 3.6 × 10^−5^ (Table S5). This is more than a fourfold difference in escape rate that could be explained by the underlying auxotrophies in BY4743 and/or the heterothallic PE-2 genome (37). Ultimately, we found that most haploid containment strains effectively escape containment by the time of the assay and have escape rates around one (Fig. 2C). However, some variability was observed in escape rate from the haploid colonies assayed. The BY haploid strains had escape rates in the range of 0.0002–1.53 (Table S5), while PE-2 haploid strains were much less variable, with escape rates from 1.15 to 1.39 (Fig. 2C; Table S5).

Additional strains were constructed to understand the biocontainment trends observed through the escape assay. For example, a haploid strain that had two copies of *relE* and one copy of cam-TA (IBY174, IBY175, & IBY176) still displayed containment rates that averaged to one, indicating complete escape by the time of the assay. In addition, a single copy diploid which contained one heterozygous copy of cam-TA and one heterozygous copy of *relE* (IBY118 & IBY119) was constructed through mating (Table S1). This strain also readily escaped and had an escape rate of one. We also noted that in diploid containment strains, increasing the number of *relE* copies did not further increase containment (Fig. 2C) (IBY137 & IBY138). Additional copies of cam-TA were added into diploid 2-copy *relE* strains to increase containment efficacy; however, mutations accumulated in the copies of cam-TA and/or *relE* before the assay, also giving escape rates of one (data not shown). Escape rate assays indicated that diploids with two homozygous copies of both cam-TA and camPr-*relE* led to the most effective containment. It was also determined that the addition of camPr-*relE* did not increase mutation rates above wildtype levels (Table S4). Given there was minimal impact on growth rates in these strains (Fig. 2; Fig. S1), we also wanted to assess if there was any compromise to bio-productivity due to the addition of biocontainment. Ethanol production was quantified through high-performance liquid chromatography to assess the impact of containment on bio-productivity. There were no significant differences in ethanol production per OD600 unit in both wildtype BY and PE-2 strains (Fig. 3). Importantly, the addition of biocontainment into the industry strain PE-2 did not have a significant impact on ethanol production compared to the wildtype counterpart (Fig. 3B and C).

To further determine escape dynamics in liquid culture, high-throughput screening in a 96-well plate was also performed. Two different BY diploid CamOff-RelE isolates (IBY69 & IBY70) were examined over the course of 72 h, and different escape frequencies were observed (Fig. S2). In the first isolate (IBY69), there was no saturation over the course of 72 h, indicating no complete escape events. However, at approximately ~68 h, wells B6, D6, and D10 began to enter logarithmic growth phase. These cells were harvested and confirmed that they grew in media that lacked camphor, indicating a true escape event. In the second isolate (IBY70), escape occurred at approximately 32 h (wells E1,F3,G4,F6, F9, H7). These cells also grew in YPD after harvesting. The different escape frequencies highlight the importance of rigorous and long-term screening to measure escape. Indeed, prior analyses of RelE efficacy indicated highly effective kill capacity at <24 h growth (30, 56).

### Whole-genome sequencing revealed independent mechanisms of escape from CamOff-RelE biocontainment

**Fig 4:**
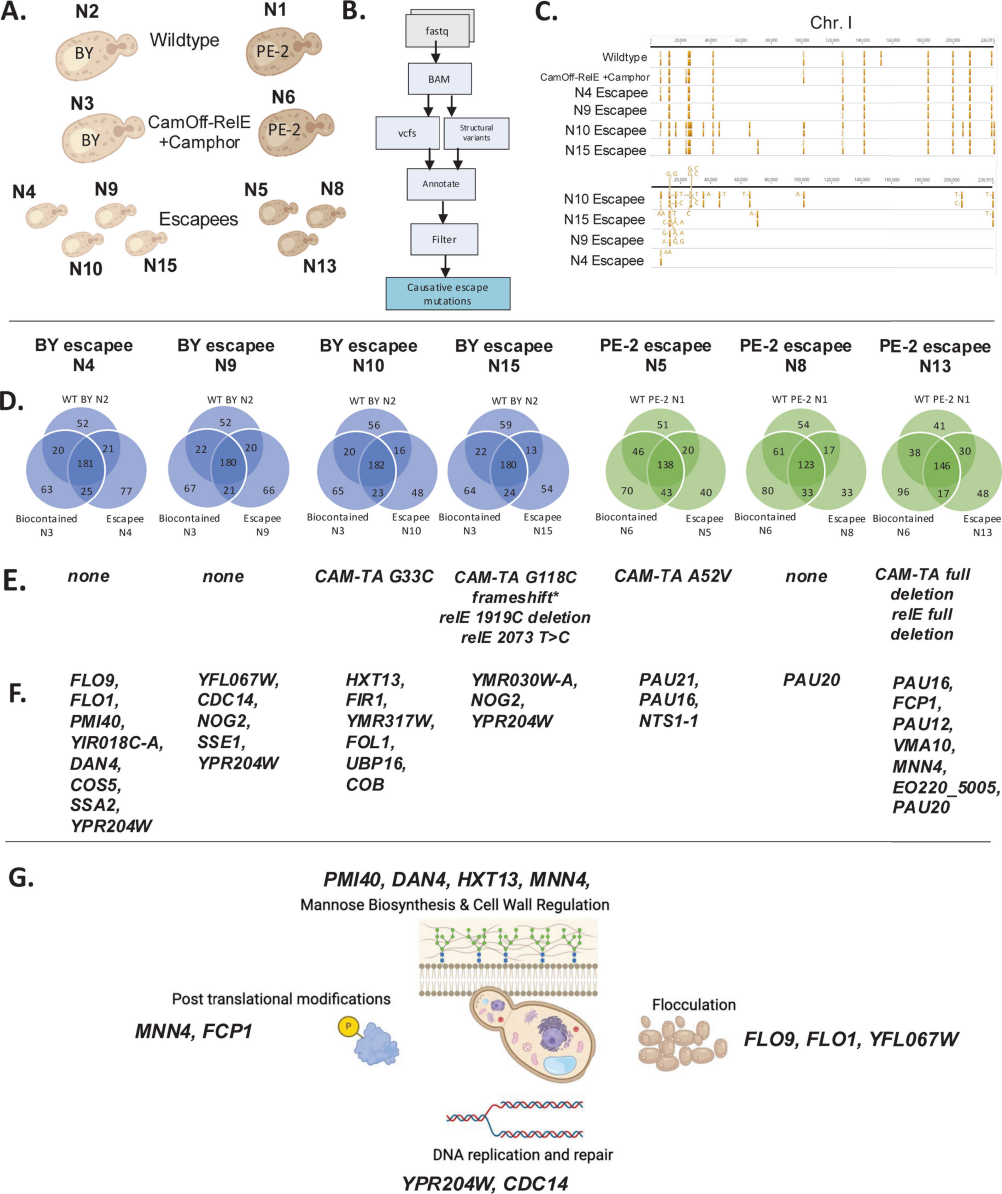
WGS reveals mutations throughout the genome that show pathway enrichment, with multiple mutations in cam-TA and RelE that could explain escape. (**A**) Sample overview for whole-genome sequencing. Full sample descriptions are supplied in Table S6. (**B**) Analysis pipeline to narrow in on causative escape mutations. (**C**) Variant filtering applied BY diploid samples. Filtering on chromosome I is displayed in Geneious Prime (Version 2025.0.3). (**D**) Variant overlap showing the number of unique genes mutated in an escapee after removing wildtype and biocontained strain mutations. (**E**) Mutations occurring in cam-TA or RelE if they are present. (**F**) Mutations found in other genes throughout the genome. (**G**) A compilation of enriched genes from SGD annotation and STRING analysis (for details, see Tables S8 and S9). The figure was created in BioRender. Lamb, N. (2025) https://BioRender.com/z69l495

Short-read whole-genome sequencing (WGS) was utilized to determine if mutations occurred in the genome of diploid escapees that could inform mechanisms of escape from CamOff-RelE biocontainment. Both the BY4743 wildtype diploid (IBY12) and PE-2 wildtype diploid (IBY2) were sequenced as parental strain references, along with BY4743 and PE-2 CamOff-RelE containment strains (IBY69 & IBY133) that had been grown in the presence of camphor (Fig. 4). A suite of seven escapees from different genotypes and growth conditions was sequenced (Table S6). The escapees sequenced came from different growth conditions, including colonies that grew up on YPD plates, which were then passaged in YPD liquid culture. It is worth noting that colonies that grew on YPD plates all had slow growth phenotypes in liquid YPD culture (data not shown). A bioinformatic pipeline was developed to remove variants in common between the wildtype and biocontainment strains and then mutated genes were annotated (detailed in Materials and Methods). We found that 4/7 sequenced escapees had mutations in either cam-TA, *relE,* or both constructs. Most frequently, the observed escape events involved loss of function mutations in our containment system, including large deletions. However, three escapees had an intact CamOff-RelE system and were able to grow in the absence of camphor. These escapees had acquired other mutations in their genomes, including in genes related to DNA replication and repair, flocculation, and post-translational modifications (Fig. 4G; Fig. S3). The mannose biosynthesis pathway was also impacted by the addition of biocontainment to these strains (Fig. 4G).

### Single point mutations in cam-TA are causative in escape

**Fig 5:**
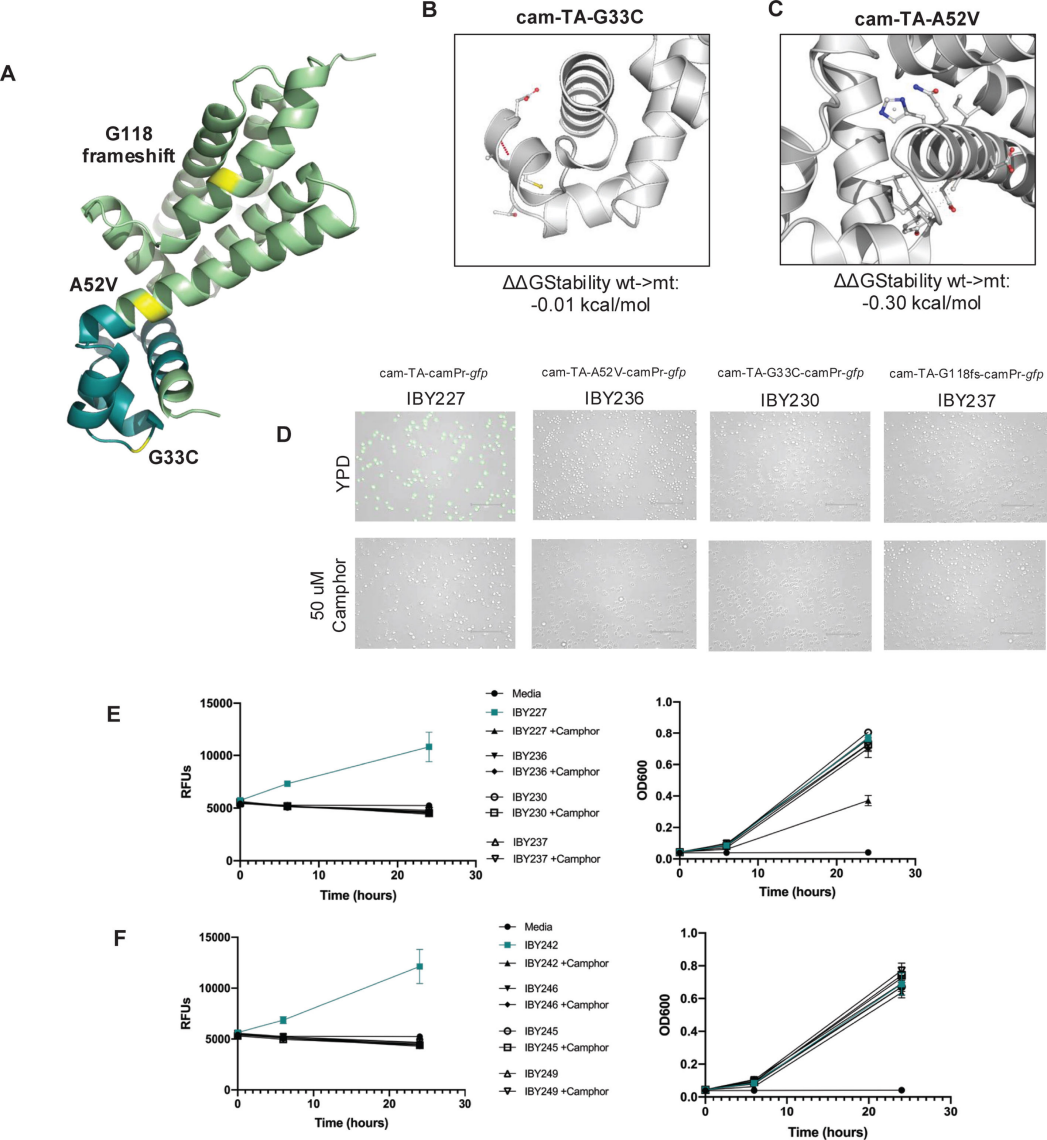
cam-TA mutations re-capitulate escape phenotypes in CamOff-GFP strains. (**A**) Alpha fold model of cam-TA, with the DNA binding domain highlighted in turquoise. The three cam-TA point mutations of interest are displayed in yellow. (**B**) The predicted impact on cam-TA structure from the G33C mutation using DDMut. (**C**) The predicted impact on cam-TA structure from the A52V mutation using DDMut. (**D**) cam-TA mutations were re-integrated into a strain containing GFP at a separate locus to see if gene expression regulation was maintained. (**E**) GFP fluorescence in haploid BY4741 strains with recapitulated cam-TA mutations and camPr-gfp measured in liquid culture over 24 h growth. (**F**) GFP fluorescence in diploid BY4743 strains with recapitulated cam-TA mutations and camPr-gfp.

A disproportionate number of mutations in cam-TA were found in escapees, both through targeted nanopore sequencing in haploids and WGS (Fig. 4; Fig. S4). Seven out of eight haploid colonies sequenced had mutations in cam-TA, while four had mutations in *relE*. Similarly, in diploid escapees four out of seven had mutations in cam-TA while two had mutations in *relE* (Fig. 4E). This was somewhat unexpected, as a nonsynonymous mutation in *relE* could lead to ineffective toxin expression. However, this finding indicates there is strong selection for cells that have acquired mutations in cam-TA. The mutations in cam-TA identified by WGS included a complete deletion of cam-TA, a two-base deletion-mediated frameshift, and two different point mutations (Fig. 4G). An alignment of the three different cam-TA mutations from WGS with the highly conserved AcrR TetR family of transcription factors (labeled COG1309) revealed that each of the three mutations occurred in highly conserved residues of cam-TA, indicating functional importance (57) (Fig. S5). Amino acid change G33C occurs in the modeled DNA-binding domain of cam-TA, likely interfering with cam-TA/camPr binding (Fig. 5A and B), while A52V is predicted to have a destabilizing impact on protein stability (ΔΔGStability wt->mt: −0.30 kcal/mol) (Fig. 5C) and the G118fs frameshift likely leads to a non-functional protein due to the introduction of a premature stop codon at amino acid residue 120.

To confirm that these mutations led to a compromise in CamOff function *in vivo*, we constructed CRISPR integration plasmids that contained cam-TA with each of the three mutations: cam-TA-G33C, A52V, and G118fs, along with a wildtype cam-TA control (Tables S1 and S2). These constructs were then integrated into strains that contained camPr-*gfp* at a different locus to assess if GFP expression was effectively regulated in the presence and absence of camphor in mutant and wildtype cam-TA backgrounds. Compared to a wildtype cam-TA-camPr-*gfp* strain grown in the absence of camphor, no fluorescent colonies were observed in any of the three cam-TA mutation strains tested (Fig. 5D). This indicates that even a single point mutation, or a small frameshift, is sufficient to confer escape in these backgrounds. Additionally, no GFP fluorescence was observed in both haploid (Fig. 5E) and diploid (Fig. 5F) strain backgrounds, indicating the loss of a functional biocontainment system from three independent mutations in cam-TA.

## DISCUSSION

While numerous studies have explored biocontainment in *S. cerevisiae,* the underlying factors affecting biocontainment efficacy remain less understood. Of note, most studies of biocontainment have focused on the use of highly domesticated laboratory strains. However, the ultimate deployment of biocontained strains would likely be in more robust, diploid, or polyploid industrial strains which differ significantly from their laboratory counterparts. Here, we present an in-depth characterization of biocontainment efficacy in laboratory and industrial strains of *S. cerevisiae* in both haploid and diploid backgrounds using the toxin RelE under control of the CamOff switch. Notably, CamOff-RelE diploid strains were contained over the course of 72 h in liquid culture (Fig. 2A and B). These diploid strains have escape rates in the range of 10^−4^ −10^−6^ when assayed on agar plates, compared to the haploid strains which have escape rates of approximately one, even when harboring two copies of the containment system (Fig. 2C). This suggests that cassette copy number alone is not sufficient to explain decreased escape frequencies in diploid cells. Furthermore, this implies that ploidy impacts biocontainment, with multiple homozygous copies of containment cassettes decreasing escape frequency.

We further explored how strains were able to escape biocontainment through genomic resequencing. Specifically, haploid strains readily escaped from the containment system and consistently had mutations within the integrated genetic constructs; either cam-TA or *relE* (Fig. S4). These haploid escapees may have also incurred additional mutations outside of cam-TA and *relE*. After transformation, colonies were screened to confirm a lack of growth in the absence of camphor; however, haploid strains had rapidly incurred mutations in cam-TA and/or *relE* even before the time they were assayed. Notably, little to no growth was observed on YPD when these strains were first transformed, indicating that escape occurred during permissive growth for the escape assays, potentially due to leakiness of the CamOff system. This further suggests a selective advantage to mutate and disrupt the function of the containment system that was introduced, highlighting that reducing generation time is a necessary consideration for effective containment both when generating and likely when using biocontained strains. Mutation accumulation experiments, starting from an initial transformation colony, could reveal the order in which the cell acquires escape mutations.

Variable escape frequencies were observed in strains with haploid genotypes in our escape assay (Fig. 2C). Variable colony sizes were also seen in haploid strains that were grown in the absence of camphor (Fig. S4A). These different sized colonies had distinct mutations in cam-TA and/or *relE* when investigated by targeted Nanopore sequencing (Fig. S4). This shows that distinct, independent, escape events occur within a single genetic isolate. Single-cell genome sequencing could be informative for determining low-frequency escape events in mixed populations over time. Likely there could be multiple pathways that lead to escape. Some WGS mutations in diploid escapees occurred at less than 100% frequency, which could be due to different populations of cells containing different mutations. However, all escape mutations found in cam-TA or *relE* in the diploid strains sent for WGS had a variant frequency of 100%, indicating both homozygous chromosomes had incurred the same mutation (Table S7). The most likely mechanism for a homozygous variant is through gene conversion of an initial mutation in one chromosome. Gene conversion commonly occurs in yeast and most likely arises through a loss of heterozygosity, followed by rapid selection (58, 59). For this kill switch, having the containment system in a homozygous fashion is favorable for decreasing escape from the CamOff-RelE system in two different strain backgrounds of *Saccharomyces cerevisiae*, both the BY series and PE-2 strains. Future work will explore whether gene conversion, thus escape, could be decreased by genetically recoding one chromosome to reduce homologous recombination.

While mutations were commonly observed in cam-TA and *relE*, three out of seven escapees sent for WGS did not incur any mutations in these constructs (Fig. 4). These escapees are of particular interest as they could likely circumvent other biocontainment approaches, including emerging gene entanglement strategies (60–62). This indicates unique pathways for escape outside of mutations in the biocontainment system introduced. Importantly, we found mutations through WGS that impacted genes in pathways related to DNA replication and repair; the phosphatase *CDC14* required for mitotic exit (63–65) and the Y’ helicase *YPR204W* (66, 67). Three distinct genes that are involved in post-translational modifications were mutated in escapees; in addition to *CDC14,* the carboxy-terminal domain phosphatase *FCP1* (68–70) and *MNN4,* which activates mannosylphosphorylation (71, 72). Together these findings lead us to hypothesize that the mutation of genes, such as those involved in DNA replication and repair and post-translational modification primes the cell for escape from CamOff-RelE containment system. Expanding the suite of escapees sequenced could further show genes that were mutated during the process of cells escaping and could highlight additional genes and pathways that could be crucial targets in preventing escape.

Through the in-depth evaluation of the CamOff-RelE system, we have learned many intricacies related to quantifying escape. Multi-day monitoring of cell growth is essential. Cells were in liquid culture for up to 70 h before any significant change in cell growth was observed (Fig. S2, IBY69). Thus, low-frequency escape events may take longer to accumulate to the point that they are detectable. We also noted that the three escapees that did not have mutations disrupting the function of cam-TA or *relE* had slower growth phenotypes than escapees with mutations in the containment constructs. The slow growth phenotype may be a precursor to detecting escape mutations in the heterologous biocontainment cassette. When performing plating assays, it was observed that certain colonies took additional days to grow up on YPD plates. It is possible that some of the smaller colonies are primed for escape but have not mutated the containment system yet. A single, slow growing escape event could take significantly longer to be observable in either liquid culture or on plates.

The above-mentioned strategies represent potential directions for better understanding containment and understanding how cells circumvent escape. Containment was achieved in diploid organisms with at least two homozygous copies of CamOff-RelE, but it is worth investigating other kill switches and toxins. Introducing RelB, the antitoxin to RelE, under an inducible promoter, could also enhance containment in this system by fully suppressing toxin activity during permissive growth, thereby addressing potential leakiness and reducing selective pressure to mutationally bypass the containment system. Additionally, modulating expression of the CamOff system through different promoters could mitigate off-target effects of the containment system, such as the elongated morphologies and the potential of chromosome condensation from intracellular camphor (44, 45). While most of this work was done in complete media (YPD or YPD with camphor), it would also be interesting to assess containment under different selective conditions. This would also allow for assessment of how variable stressors and conditions contribute to escape under laboratory conditions. To further assess escape mechanisms, these containment strains can be tested in a mock-environmental release situation through the use of a terrestrial mesocosm system (12). It is of particular interest if auxotrophies found within the BY series of strains contribute to effective biocontainment under environmental release conditions.

The integration of the CamOff-RelE system into distinct strains of *S. cerevisiae* highlighted critical considerations for biocontainment design, particularly in the development of kill switches. Ploidy is an essential factor for achieving effective containment with CamOff-RelE in both lab and industrial strains of yeast. These findings may be highly applicable to biocontainment in eukaryotic organisms with multiploidy, including other yeast species, protozoa, and algae (73) although the effectiveness of DNA repair mechanisms in each strain may affect varied escape frequency. It remains to be tested if vastly different genomes may lead to different mechanisms of escape from the containment system. Notably, we observed significant differences in escape rates between the BY diploid CamOff-RelE strains and the PE-2 diploid CamOff-RelE strains (Fig. 2C). This system may also have variable biocontainment efficacy when integrated into other fungi species. BY diploid strains acquired different mutations in cam-TA or *relE* than were seen in the PE-2 diploid background. This indicates that different strain backgrounds of *S. cerevisiae* may be primed differently for escape from the containment system, in part, due to underlying auxotrophies in BY strains and the heterothallism of PE-2-derived strains. A critical finding in this study is that the CamOff-RelE kill switch is effective in an industrially relevant strain of yeast (PE-2) with minimal compromise to biocatalytic productivity. This is a crucial finding for adopting biocontainment for GMMs grown at scale (9) and designing safe-guarded strains that focus on the introduction of biocontainment modules that are stably maintained in the genome. However, additional studies of CamOff-RelE strains grown at industry-scale volumes will be crucial to understanding escape dynamics in larger cultures before the CamOff-RelE switch is utilized in industry as a means of biocontainment. Altogether, our findings highlight important conclusions that can be applied to successful kill switch deployment as a means of biocontainment and future considerations for secure biosystems design.

## MATERIALS AND METHODS

### Yeast strain construction and culture conditions

Yeast strains were constructed in two parental backgrounds: the BY lab series and the industrially relevant PE-2 background. The BY4741 haploid and the BY4743 diploid were utilized, as well as the JAY291 haploid and JAY270 diploid PE-2 strains. BY4741 and BY4743 strains were purchased from ATCC. PE-2 strains were provided by Juan Lucas Argueso (37). These strains were modified to contain biocontainment constructs through the CRISPR Easyclone Marker Free system, as previously described (43). Integration plasmids were cloned to contain *TDH3*pr-cam-TA, camPr-*gfp*, and camPr-*relE* (Tables S1 to S3). *TDH3*pr-cam-TA was purchased as a gBlock from Twist and integrated into Easyclone plasmids following standard procedure (43). All integrations were confirmed via PCR, followed by Nanopore sequencing (Plasmidsaurus) at the integration site (Fig. S1). Yeast strains are displayed in Table S1 and plasmids are displayed in Table S2. Liquid YPD media was composed of 10 g yeast extract and 20 g peptone per liter of media with D-glucose at 2% final concentration. YPD agar plates were made from YPD + Agar mix, added at 65 g/L (Sigma Y1500). Camphor stocks were made at a concentration of 100 mM in DMSO. Camphor stocks were kept at −20°C, away from light and used only once after thawing. 100% DMSO was utilized as a control in growth studies that utilized camphor.

### Microscopy

Microscopy was performed on cells grown on YPD or YPD with 50 µM camphor plates. Cells were resuspended in 10 µL of 1× TE (10 mM Tris-HCl, pH 7.4; 1 mM EDTA). Slides were imaged at both 40× and 100× on an EVOS M5000 microscope (Fisher Scientific), using both *trans* light and through a GFP filter cube. *Trans* and GFP overlayed images are displayed in Fig. 1 and 5.

### Liquid growth screening

Growth in liquid culture was screened using a BioTek LogPhase 600 microbiology plate reader (Agilent). Corning 96-well clear flat bottom plates (Corning CLS3598) were inoculated with 150 µL total volume per well and sealed with a clear plate seal (TopSeal A-Plus). Cells were grown in three technical replicates, leaving a border of media around the outside edge of the plate to account for any evaporation. Camphor was added at a final concentration of 25 µM and 50 µM. DMSO was added to YPD controls at 1% total volume. The plates were incubated at 30°C, with shaking at 800 rpm, and OD600 readings were taken every 10 min over 72 h. Seventy-two hours was sufficient time to observe saturation of wildtype strains, as well as observe escape events from biocontained strains. Max Rate (OD/min) was calculated via the LogPhase 600 and defined as the maximum slope of the growth curve generated. Lag Time was also quantified by the LogPhase 600, as the time intercept between the Max Rate line and the horizontal line that passes through the first point. End point OD600 is also reported, which is the final OD600 reading reached after 72 h growth (Fig. S1).

### Canavanine resistance assay

Canavanine resistance assays were performed to assess mutation rates at the *CAN1* locus. Two-millimeter colonies from YPD or YPD + 50 µM camphor plates were selected to assay. Colonies were suspended in 100 µL of 1×TE buffer pH 7.4. Resuspended colonies were then diluted 10^−4^. Seventy-five microliters of the undilute colony suspension was plated on half of a −ARG + Canavanine plate or −ARG + Canavanine + 50 µM camphor plate. Twenty microliters of the 10^−4^ dilution was plated on a half plate of −ARG or −ARG + 50 µM camphor. Colonies were counted after reaching approximately 1 mm in size. Mutation rates were calculated using fluctuation analysis through Flucalc. Flucalc uses the Ma-Sandri-Sarkar maximum likelihood estimation (MSS-MLE) method (74) to determine mutation rates and calculate 95% confidence intervals. At minimum, 19 colonies were assayed per genotype. When available, at least two isolates were assayed per genotype.

### HPLC quantification of ethanol

Cells were conditioned to 0.5× YEP (Yeast Extract: Sigma Aldrich Y1625, Bacto Peptone: Thermo Fisher Scientific 211677) with 10% glucose in 5 mL overnights, pre-inoculation. Twenty-five milliliters of cultures of 0.5× YEP + 10% glucose were then inoculated at a 1:1,000 dilution and grown in anaerobic bottles, in triplicate. One milliliter of samples was taken from cell culture and filtered through MDI SY25GN 0.2 µm filters into 1.5 mL glass crimp micro vials. An Agilent 1260 Infinity II HPLC system and an Agilent 1260 Infinity II Refractive Index Detector were used for HPLC analysis. LC separation was performed using an Aminex HPX-87H chromatographic column at 55°C, with 0.01N sulfuric acid aqueous solution as mobile phase. The flow rate was 0.6 mL/min, and the injection volume was 6 µL per sample. For calibration, ethanol standards were purchased from Absolute Standards Inc. Post-run data acquisition and analysis were performed using an Agilent Chemstation. Ethanol quantification is reported in g/mL.

### Escape rate assays

To quantify escape rate, a reversion plating assay was performed, similar to previously described work (26). Briefly, a strain of interest was struck onto YPD with 25 µM camphor and grown at 30°C until 2 mm single colonies formed. A single 2 mm colony was then inoculated into 5 mL of YPD with 25 µM camphor media and allowed to grow overnight (~18 h). Each saturated 5 mL culture was then pelleted and washed twice, with two volumes of sterile water to remove camphor. The cell pellet was resuspended in 500 µL sterile water and diluted to 10^−5^. Spot assays and initial test assays were performed to determine the proper dilution range for specific genotypes. The 10^−4^ and 10^−5^ dilutions were plated onto YPD + 50 µM camphor. These plates were then incubated at 30°C for 3–4 days. The YPD plates with escapees were left at 30°C for a maximum of 7 days, as we observed variable colony sizes and growth from our escape assay, likely highlighting that different genetic mutations are represented on one of these plates. At minimum, three biological replicates were assayed and escape rates were determined using the method of the median. The median escape rate and 95% confidence intervals were calculated in Prism (version 9.0.2).

### Whole-genome sequencing and bioinformatics

Cell pellets were sent to Azenta for short-read Illumina, 2 × 250, whole-genome sequencing (WGS). Azenta performed initial bioinformatic processing, including quality and adapter trimming using Trimmomatic v.0.36. Reads were aligned to the S288C reference genome (https://www.ncbi.nlm.nih.gov/datasets/genome/GCF_000146045.2/) and PE-2 reference genome (https://www.ncbi.nlm.nih.gov/datasets/genome/GCA_905220325.1/) with separate reference genomes for RelE or cam-TA constructs in the appropriate genomic location (https://github.com/nalamb/Mutation-Filtering-in-S.-cerevisiae), through Burrows-Wheeler Aligner (BWA v.0.7.12). Variants including small-nucleotide variants (SNVs) and small insertion/deletions (INDELs), along with variant frequency, were determined using mpileup and VarScan v.2.3.9. Variant frequency is determined by VarScan, as the total number of sequencing reads that contain a specific base that differs from the reference genome at a particular genomic position, relative to the sequencing depth at that position. Structural variants (SVs) were annotated using Manta v.1.4.0. Variant files were then annotated through the bcftools annotate function, using gene reference files in BED format. A python program was then developed to filter out common variants between wildtype and an escaped strain utilizing vcf files. This filter was used to determine variants that would be most causative in explaining escape. Filtering code is available via Github (https://github.com/nalamb/Mutation-Filtering-in-S.-cerevisiae). Sequencing data is available on SRA (https://www.ncbi.nlm.nih.gov/bioproject/1187634).

### Cam-TA mutation characterization

The CamR structure was modeled using a previously published alpha fold model of the Cam repressor from *Pseudomonas putida* (accession: M5AWW0). CamR figures were generated in PyMOL Version 2.5.4. The impact of G33C and A52V mutations on CamR structure was evaluated through DDMut (75). Protein sequence alignments (Fig. S5) were made using Clustal Omega Multiple Sequence Alignment (MSA). The alignment shown uses the consensus sequence of COG1309 (https://www.ncbi.nlm.nih.gov/Structure/cdd/COG1309). Cam-TA mutations were cloned into Easyclone integration plasmids (Table S2) following standard methods (43). The cam-TA constructs were then integrated with camPr-*gfp* in the BY4741 and BY4743 strain backgrounds (Table S1). GFP fluorescence was measured from liquid culture using a Tecan infinite 200Pro with Tecan i-control software (version 2.0.10.0), with the excitation wavelength set at 485 nm and emission at 535 nm. Strains were grown in triplicate and the mean of three technical replicates was plotted using Prism (version 9.0.2).

## Data Availability

All sequencing data in the form of fastq files are available on SRA: PRJNA1187634. Mutation filtering code can be found on Github: https://github.com/nalamb/Mutation-Filtering-in-S.-cerevisiae. All variants sequenced from WGS are displayed in Table S7. All structural variants are displayed in Table S8. STRING analysis can be found in Table S9. Mutated genes of interest are found in Table S10.

## SUPPLEMENTAL MATERIAL

Fig. S1 aem.00741-25-s0001.eps Liquid growth screening of addition of cam-TA and GFP shows minimal growth impact.

Fig. S2 aem.00741-25-s0002.eps High throughput escape monitoring of diploids.

Fig. S3 aem.00741-25-s0003.eps STRING analysis showing networks of mutated genes.

Fig. S4 aem.00741-25-s0004.eps Haploid sequencing revealed distinct mutations in cam-TA and/or RelE.

Fig. S5 aem.00741-25-s0005.eps Alignment of cam-TA with AcrR model for transcription factors (COG1309), highlighting conserved residues.

Supplemental tables aem.00741-25-s0006.xlsx Tables S1 to S10.

ASM does not own the copyrights to Supplemental Material that may be linked to, or accessed through, an article. The authors have granted ASM a non-exclusive, world-wide license to publish the Supplemental Material files. Please contact the corresponding author directly for reuse.
